# Utility of the non-high-density lipoprotein cholesterol to high-density lipoprotein cholesterol ratio in individuals with retinal vein occlusion

**DOI:** 10.3389/fcvm.2025.1692351

**Published:** 2025-12-16

**Authors:** Kaichao Xia, Yang Yang, Ziyan Song, Yiqiao Xing, Anhuai Yang, Kaibao Ji

**Affiliations:** Department of Ophthalmology, Renmin Hospital of Wuhan University, Wuhan, Hubei, China

**Keywords:** atherosclerosis, lipid marker, NHHR, retinal vein occlusion, risk factor

## Abstract

**Purpose:**

The ratio of non-high-density lipoprotein cholesterol to high-density lipoprotein cholesterol (NHHR) has recently been identified as a novel lipid marker for assessing the risk of atherosclerosis-related diseases. However, the relationship between NHHR and the risk of retinal vein occlusion (RVO) has not yet been thoroughly investigated. The objective of this study was to investigate the correlation between NHHR and patients with RVO.

**Methods:**

This retrospective study examined 54 patients diagnosed with RVO and 57 age- and gender-matched control subjects. Comprehensive ocular examinations and hematological assessments were conducted for all participants. Logistic regression analysis was employed to evaluate the association between lipid markers and the risk of RVO. The receiver operating characteristic (ROC) curve was utilized to analyze and determine the predictive value and optimal threshold of the NHHR, triglyceride-glucose (TyG) index, and other conventional lipid parameters for RVO.

**Results:**

Compared to the control group, patients with RVO exhibited significantly higher levels of triglyceride (TG), TyG index, and NHHR (*P* = 0.0004, *P* = 0.0006, and *P* < 0.0001, respectively). Additionally, the high-density lipoprotein cholesterol (HDL-C) index was significantly lower in the RVO group compared to the control group (*P* < 0.0001). Univariate analysis indicated that NHHR (OR: 3.41, *P* < 0.001), TyG index (OR: 3.32, *P* = 0.001) and TG (OR: 2.64, *P* = 0.003) were significantly associated with RVO. Multivariate analysis revealed that NHHR was remarkably associated with RVO (OR: 2.09, *P* = 0.037). After further adjustment for hypertension, TG, and the TyG index, this association remained statistically significant (OR: 3.13, *P* = 0.003). The areas under the ROC curve for TyG index, TG, HDL-C, and NHHR were 0.679, 0.692, 0.739, and 0.752, respectively. Notably, the AUC value for NHHR demonstrated a moderate sensitivity (50.88%) and high specificity (87.04%), indicating its potential as a promising biomarker for the diagnosis and prognosis of RVO.

**Conclusion:**

The NHHR was significantly elevated in patients with RVO, suggesting that this novel lipid marker may play a crucial role in the risk of developing RVO.

## Introduction

Retinal vein occlusion (RVO) represents the second most prevalent retinal vascular lesion following diabetic retinopathy, impacting approximately 28.06 million individuals globally in 2015 (1, 2). According to the anatomic occlusion location, RVO can be divided into branch RVO (BRVO) with a prevalence of 0.6%–1.6% and central RVO (CRVO) affecting 0.1%–0.4% (1). A multitude of acknowledged risk factors, including advanced age, hypertension, diabetes mellitus, hypercholesterolemia, smoking, elevated body mass index, hypermetropia, ocular conditions such as intraocular hypertension and glaucoma, as well as orbital diseases, have been identified as contributors to this condition (3, 4). The exact etiopathogenesis of RVO remains not fully elucidated. Prior research has suggested that the primary pathogenetic mechanisms in most cases involve the compression and mechanical stenosis of the retinal vein due to an atherosclerotic artery, as well as local inflammation induced by venous stasis and exudation (5, 6). Furthermore, individuals with systemic arteriosclerotic vascular disorder are at a significantly increased risk of developing this condition (7).

Dysregulated lipid metabolism, marked by elevated levels of low-density lipoprotein cholesterol (LDL-C), LDL-triglycerides, and lipoprotein(a) [LP(a)], may play a significant role in the pathogenesis of retinal vascular occlusions (8). High-density lipoprotein cholesterol (HDL-C), characterized by its small and dense lipoprotein particles, has been shown to exert protective effects against atherosclerosis (9). Conversely, non-high-density lipoprotein cholesterol (non-HDL-C), which encompasses LDL-C, intermediate-density lipoprotein (IDL), LP(a), and remnants of very low-density lipoprotein (VLDL), contributes significantly to the progression of atherosclerosis (10, 11). Furthermore, there is a significant correlation between the Triglyceride-glucose (TyG) index and cardiovascular diseases associated with atherosclerosis (12). In recent years, the clinical significance of non-HDL cholesterol (non-HDL-C) has garnered considerable attention and recognition in the medical community. In 2021, the UK-based National Institute for Health and Care Excellence (NICE) issued guidelines recommending non-HDL-C as the primary target for mitigating cardiovascular disease risk in diabetic patients, superseding LDL-C (13). This shift underscores the importance of non-HDL-C in evaluating cardiovascular risk and treatment efficacy (14). However, a novel composite lipid indicator, the ratio of non-HDL-C to HDL-C (NHHR), has emerged as an advanced metric for comprehensive lipid assessment, encompassing both pro-atherogenic and anti-atherogenic lipid particles (15). Scholarly research has demonstrated that NHHR exhibits superior predictive power compared to traditional lipid parameters for assessing the risk of atherosclerosis and cardiovascular disorders (16, 17).

However, the relationship between NHHR and RVO remains underexplored. To address this gap, this study aims to investigate the correlation between NHHR and the incidence of RVO, thereby providing valuable insights for disease management and prevention strategies.

## Methods

### Study population and design

This study was approved by the Ethics Review Committee of Renmin Hospital of Wuhan University (WDRY2022-K278) and conducted in accordance with the tenets of the Declaration of Helsinki. Due to the retrospective nature of this study and the absence of any identifiable patient information, the Ethics Committee of Renmin Hospital of Wuhan University waived the requirement for individual informed consent. Medical records of patients diagnosed with RVO at the Department of Ophthalmology, Renmin Hospital of Wuhan University, between October 2023 and January 2025 were systematically reviewed and analyzed. Data for the control group were derived from individuals who underwent cataract surgery at our hospital during the same timeframe and were otherwise healthy.

The inclusion criteria for patients with RVO were based on characteristic clinical features such as flame-shaped and dot-blot hemorrhages, edema, cotton-wool spots, hard exudates, venous dilation, tortuosity, and findings from fundus fluorescein angiography (FFA). Patients were excluded if they had any of the following conditions: (1) a history of other ocular disorders, including keratoconjunctivitis, glaucoma, uveitis, scleritis, or any associated retinal vascular disorders; (2) a history of intraocular surgery; or (3) any systemic disease other than hypertension, diabetes mellitus, and hyperlipidemia. The control group consisted of age- and sex-matched patients with cataracts who did not have any ocular complications, a history of intraocular surgery, or systemic diseases, except for hypertension, diabetes mellitus, and hyperlipidemia.

### Data collection

All participants underwent a comprehensive ophthalmologic examination, which included assessment of best-corrected visual acuity (BCVA), measurement of intraocular pressure (IOP), slit-lamp biomicroscopy, color fundus photography, spectral-domain optical coherence tomography (SD-OCT), and FFA. Peripheral blood samples were collected from all participants on the morning of the second day following the initial diagnosis of RVO, after an 8–12 h overnight fast, to assess hematologic parameters.

The levels of fasting blood glucose, total cholesterol (TC), triglycerides (TG), high-density lipoprotein cholesterol (HDL-C), and low-density lipoprotein cholesterol (LDL-C) were meticulously measured using an advanced automatic biochemical analyzer (ADVIA® Chemistry XPT System, Tokyo, Japan). Remnant cholesterol (RC) was derived by subtracting HDL-C and LDL-C from TC. Non-HDL-C was determined by subtracting HDL-C from TC. The TyG index was computed using the formula ln [fasting triglycerides (mg/dL) × fasting glucose (mg/dL)/2] (18). The NHHR was established by calculating the ratio of non-HDL-C to HDL-C (19).

### Statistical analysis

All statistical analyses were conducted using GraphPad Prism 9.0 (GraphPad Software, San Diego, CA, USA) and IBM SPSS Statistics 22.0 (IBM Corporation, Armonk, NY, USA). The normality of data distribution was evaluated using the Shapiro–Wilk test. Normally distributed continuous variables are presented as mean and standard deviation (SD), whereas non-normally distributed data are reported as median with interquartile range (IQR). Comparisons between groups for continuous variables were performed using independent sample t-test or Mann–Whitney *U* test, depending on the distribution characteristics. Categorical variables are summarized as frequency and percentage, and differences between groups were assessed using the Chi-square test. Logistic regression analysis was utilized to investigate the factors influencing RVO. Receiver operating characteristic (ROC) curve analysis was conducted to assess the predictive ability of potential biomarkers. The optimal cut-off value was identified using the Youden index. A *P*-value < 0.05 was considered statistically significant.

## Results

### Baseline characteristics of participants

A total of 54 patients diagnosed with RVO and 57 age- and gender-matched control subjects were enrolled in this study. Demographic characteristics of participants are summarized in Table 1. The median ages for the RVO group and the control group were 60.0 years and 65.0 years, respectively (*P* = 0.106). The incidence of hypertension was significantly higher in the RVO group compared to the control group (*P* = 0.028).

**Table 1:** The baseline characteristics of participants in the RVO group and the control group are presented.

Characteristics	RVO (*n* = 54)	Control (*n* = 57)	*P* value
Age (years)	60.0 (55.0, 70.0)	65.0 (59.0, 72.5)	0.106^a^
Gender (Male/Female)	23/31	20/37	0.417^b^
Hypertension (*n*, %)	29 (53.70%)	15 (26.32%)	0.003^b^
Diabetes mellitus (*n*, %)	9 (16.67%)	9 (16.07%)	0.933^b^
Glucose (mmol/L)	5.10 (4.75, 5.50)	4.95 (4.62, 5.69)	0.425^a^
TC (mmol/L)	4.58 ± 0.75	4.67 ± 0.94	0.577^c^
TG (mmol/L)	1.54 (1.13, 2.08)	1.22 (0.84, 1.62)	0.0004^a^
HDL-C (mmol/L)	1.10 (0.93, 1.22)	1.31 (1.11, 1.63)	<0.0001^a^
LDL-C (mmol/L)	2.68 ± 0.70	2.57 ± 0.74	0.442^c^
RC (mmol/L)	0.67 (0.56, 0.93)	0.71 (0.58, 0.90)	0.898^a^
non-HDL-C (mmol/L)	3.48 ± 0.68	3.30 ± 0.80	0.185^c^
TyG index	8.85 ± 0.58	8.46 ± 0.57	0.0006^c^
NHHR	3.22 (2.58, 3.81)	2.37 (1.94, 3.13)	<0.0001^a^

RVO, retinal vein occlusion; TC, total cholesterol; TG, triglyceride; HDL-C, high-density lipoprotein cholesterol; LDL-C, low-density lipoprotein cholesterol; RC, remnant cholesterol; non-HDL, non-high-density lipoprotein cholesterol; TyG index, triglyceride-glucose index; NHHR, non-high-density lipoprotein cholesterol to high-density lipoprotein cholesterol ratio.

^a^ Mann–Whitney test.

^b^ Chi-Square test.

^c^ Independent sample *t*-test.

**Figure 1:**
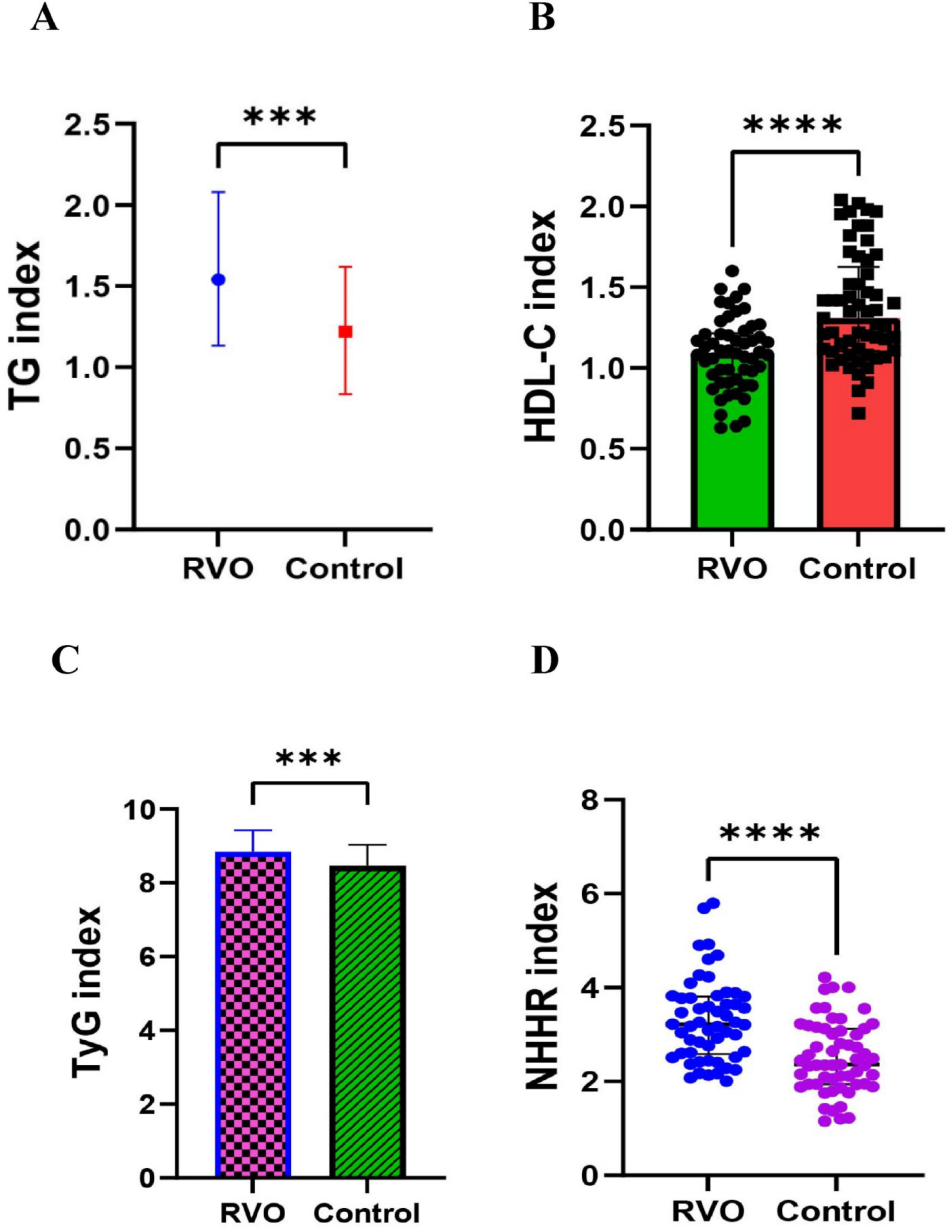
Differences in metabolic parameters between the RVO and control group. Metrics include: **(A)** Triglyceride (TG); **(B)** High-Density Lipoprotein Cholesterol (HDL-C); **(C)** Triglyceride-Glucose Index (TyG); **(D)** Non-High-Density Lipoprotein Cholesterol to High-Density Lipoprotein Cholesterol Ratio (NHHR).

No significant differences were observed between the RVO and control groups regarding fasting blood glucose (*P* = 0.425), TC (*P* = 0.577), LDL-C (*P* = 0.442), RC (*P* = 0.898), and non-HDL-C (*P* = 0.185), as detailed in Table 1. Conversely, TG, the HDL-C, TyG index, and NHHR values exhibited significant differences between the RVO and control groups, as depicted in Figures 1A–D.

### Associations between four indices and RVO

Univariate and multivariate analyses were conducted to examine the risk factors distinguishing the RVO group from the control group, as showed in Table 2. Univariate analysis demonstrated that NHHR had the highest odds ratio (OR = 3.41, *P* < 0.001), followed by the TyG index (OR = 3.32, *P* = 0.001), TG (OR = 2.64, *P* = 0.003), and HDL-C (OR = 0.03, *P* < 0.001). Multivariate analysis revealed that NHHR was remarkably associated with RVO (OR = 2.09, *P* = 0.037), as was HDL-C (OR = 0.1, *P* = 0.031), as presented in Table 2. Elevated serum NHHR, TyG index, and TG levels were identified as risk factors for RVO, while higher HDL-C levels reduced the risk of RVO. After adjusting for age, gender, hypertension, TG, and TyG index in Model 3, the association between NHHR and RVO remained statistically significant (OR = 3.13, *P* = 0.003), as shown in Table 3.

**Table 2:** Univariate and multivariate analyses were performed to identify factors linked to RVO.

Variables	Univariate analysis					Multivariate analysis				
	*β*	SE	Z	*P* value	OR (95%CI)	*β*	SE	Z	*P* value	OR (95%CI)
Gender										
M					1.00 (Reference)					
F	−0.32	0.39	−0.81	0.418	0.73 (0.34–1.57)					
Hypertension										
No					1.00 (Reference)					
Yes	1.18	0.41	2.90	0.004	3.25 (1.47–7.20)					
Diabetes mellitus										
NO					1.00 (Reference)					
YES	0.06	0.52	0.13	0.900	1.07 (0.39–2.93)					
Age	−0.04	0.02	−1.75	0.080	0.96 (0.92–1.00)					
Glucose	0.04	0.14	0.28	0.779	1.04 (0.79–1.37)					
TC	−0.13	0.22	−0.56	0.573	0.88 (0.57–1.37)					
TG	0.97	0.32	2.99	0.003	2.64 (1.40–4.99)					
HDL-C	−3.65	0.89	−4.10	<0.001	0.03 (0.00–0.15)	−2.28	1.06	−2.16	0.031	0.10 (0.01–0.81)
LDL-C	0.21	0.27	0.77	0.439	1.23 (0.73–2.08)					
non-HDL-C	0.35	0.26	1.33	0.185	1.41 (0.85–2.36)					
RC	0.56	0.52	1.09	0.277	1.76 (0.64–4.87)					
NHHR	1.23	0.29	4.19	<0.001	3.41 (1.92–6.05)	0.74	0.35	2.09	0.037	2.09 (1.05–4.18)
TyG index	1.20	0.38	3.19	0.001	3.32 (1.59–6.94)					

RVO, retinal vein occlusion; TC, total cholesterol; TG, triglyceride; HDL-C, high-density lipoprotein cholesterol; LDL-C, low-density lipoprotein cholesterol; non-HDL, non-high-density lipoprotein cholesterol; RC, remnant cholesterol; NHHR, non-high-density lipoprotein cholesterol to high-density lipoprotein cholesterol ratio; TyG index, triglyceride-glucose index; OR, odds ratio; CI, confidence interval.

**Table 3:** Adjusted logistic regression model for the association between NHHR and RVO.

Variables	Model 1		Model 2		Model 3	
	OR (95%CI)	*P*	OR (95%CI)	*P*	OR (95%CI)	*P*
NHHR	3.41 (1.92∼ 6.05)	<.001	3.05 (1.69–5.50)	<.001	3.13 (1.48–6.65)	0.003

OR, odds ratio; CI, confidence interval.

Model 1: No adjustment.

Model 2: Adjusted for Age, Gender, and Hypertension.

Model 3: Adjusted for Age, Gender, Hypertension, TG, and TyG index.

### Predictive value of each index for RVO

**Figure 2:**
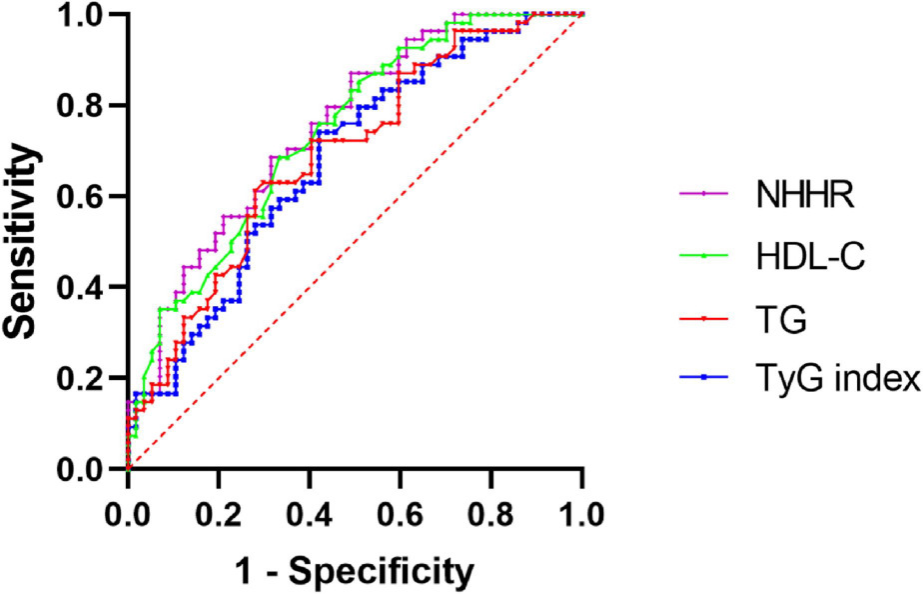
Receiver operating characteristic (ROC) curves for TyG index, TG, NHHR, and HDL-C in predicting RVO. NHHR, non-high-density lipoprotein cholesterol to high-density lipoprotein cholesterol ratio; HDL-C, high-density lipoprotein cholesterol; TG, triglyceride; TyG index, triglyceride-glucose index; RVO, retinal vein occlusion.

ROC curves for TG, TyG, NHHR, and HDL-C are presented in Figure 2. The optimal cutoff values and AUC values, along with their corresponding sensitivity and specificity, are summarized in Table 4. Among the entire study population, NHHR exhibited the highest AUC value (AUC = 0.752; 95%CI: 0.663–0.841), followed by HDL-C (AUC = 0.739; 95%CI: 0.649–0.830), TG (AUC = 0.692; 95%CI: 0.594–0.789), and TyG index (AUC = 0.679; 95%CI: 0.580–0.778). Notably, NHHR exhibited a moderate sensitivity (50.88%) and high specificity (87.04%), highlighting its potential as a promising biomarker for diagnosing and predicting RVO.

**Table 4:** Results under the receiver operating characteristic curves for each index in the identification of RVO.

Index	AUC	95% CI	*P* value	Cutoff value	Sensitivity	Specificity (%)
NHHR	0.752	0.663–0.841	<0.0001	2.37	50.88	87.04
HDL-C	0.739	0.649–0.830	<0.0001	1.18	66.67	68.52
TG	0.692	0.594–0.789	0.000	1.42	70.18	62.96
TyG index	0.679	0.580–0.778	0.001	8.48	57.89	74.07

RVO, retinal vein occlusion; AUC, area under the curve; CI, confidence interval; NHHR, non-high-density lipoprotein cholesterol to high-density lipoprotein cholesterol ratio; HDL-C, high-density lipoprotein cholesterol; TG, triglyceride; TyG index, triglyceride-glucose index.

## Discussion

Comprehensive evaluation of hematologic parameters in patients with retinal vein occlusion can enhance our understanding of the disease and aid in more accurate diagnosis and treatment. In the present study, we investigated NHHR, a newly identified atherosclerosis biomarker in patients with retinal vein occlusion. Compared with control subjects, individuals with RVO were found to have significantly higher NHHR levels. Additionally, through ROC analysis, we established that NHHR could serve as an independent risk factor for RVO incidence in the general population, distinct from traditional clinical information.

RVO is the second most prevalent cause of severe vision loss after diabetic retinopathy. Its exact pathogenic mechanisms need further elaboration and has been a subject of considerable debate in the literature. Arteriosclerotic alterations play a critical role in the etiopathogenesis of RVO. Strong associations have been identified in most cases, involving compression and mechanical stenosis of the retinal vein caused by an atherosclerotic artery, as well as focal vascular inflammation (5, 6). The occluded area undergoes a pathological progression characterized by degenerative changes in the vessel walls, atherosclerosis, abnormal blood constituents, and impaired blood flow (20). A cycle of exacerbation may be initiated at the occlusion site, where reduced flow leads to increased blood viscosity, subsequently resulting in further flow reduction (20).

Abnormal blood lipid compositions have been identified as critical mediators that contribute to the promotion of atherosclerotic lesions. Study data revealed that, among individuals with low to moderate cardiovascular risk, high TG was related with subclinical atherosclerosis and vascular inflammation, even in subjects with normal LDL-C levels (21). TG is a major component of triglyceride-rich lipoproteins (TRLs), which include chylomicrons, VLDL, and their remnants generated during TG metabolism (22). TRL remnants carry more cholesterol per particle than LDL due to their larger size and do not require modification or oxidation to become atherogenic, allowing them to be directly taken up by macrophages. Consequently, compared with LDL, TRL remnants may exhibit a stronger atherogenic potential (22). In this study, elevated TG levels were observed in patients with RVO, suggesting that TG may play a role in the pathogenesis of RVO. A previous study demonstrated that TG levels were significantly higher in patients with BRVO and CRVO compared to the control group (23). The TyG index, a valuable biomarker, has emerged as a reliable indicator of atherosclerosis in recent years (24). Previous study has demonstrated that a higher TyG index may be independently associated with an increased incidence of atherosclerosis-related cardiovascular disorders (25). Hypertension is a well-established contributing factor to atherosclerosis, which is closely linked to the prevalence of BRVO (26). A large-scale study involving 492,488 patients revealed that hypertension was the primary driver of elevated risk for BRVO events, with more severe hypertension further exacerbating this risk (27). Hypertension plays a critical role in the pathogenesis of atherosclerosis. The hardening of retinal arteries leads to compression of adjacent retinal vessels within the adventitial sheath, resulting in venous stasis and increasing susceptibility to thrombosis (28). Numerous prospective cohort studies have confirmed the robust association between the TyG index and hypertension (29, 30). Our research found that TyG index values were significantly higher in the RVO patient group compared to the control group, potentially contributing to the development of RVO through the initiation of atherogenic processes. Second, the TyG index is widely recognized as a reliable marker of insulin resistance (31), and previous studies have demonstrated its superior performance compared to the homeostatic model assessment in detecting insulin resistance (32), with high sensitivity (96.5%) and specificity (85.0%) (33). Insulin resistance has been associated with persistent low-grade systemic inflammation (34) and hyperglycemia-induced tissue damage (35). Moreover, when TG levels are markedly elevated, TG-rich chylomicrons become too large to traverse the endothelial barrier and infiltrate the arterial intima, contributing to the development of atherosclerosis (36). Third, insulin resistance may directly induce endothelial dysfunction, which is a key pathophysiological mechanism underlying the initiation and progression of atherosclerosis (25).

The NHHR, a newly lipid parameter, has emerged as an advanced metric for comprehensive lipid assessment, including both pro-atherogenic and anti-atherogenic lipid components (15). NHHR is calculated as the ratio of non-HDL-C to HDL-C, offering a cost-effective and readily obtainable measure. Non-HDL-C, which includes LDL-C and other atherogenic particles, contributes to the pathophysiology of atherosclerosis (37). Conversely, HDL-C exhibits anti-inflammatory, antioxidant, and anti-atherogenic properties, showing a negative correlation with the incidence of atherosclerotic cardiovascular diseases (38). In this study, our results indicate that NHHR values were significantly elevated in the RVO group compared to the control group, with an AUC value of 0.752. These findings suggest that higher NHHR levels may contribute to the development of RVO. Furthermore, reduced levels of HDL-C were observed in patients with RVO, which aligns with findings from several prior studies (39, 40). The NHHR is an innovative composite marker for assessing the lipid profile associated with atherosclerosis risk. Elevated NHHR indicates increased levels of non-HDL cholesterol, particularly LDL-C, which is susceptible to oxidation, leading to the formation of oxidized LDL (ox-LDL). Ox-LDL is highly atherogenic and initiates oxidative stress responses by activating the c-Jun N-terminal kinases (JNK)1/2 inflammatory signaling pathway, Lyn and mitogen-activated protein kinase (MAPK)/ERK kinase family proteins (MEKK), and protein kinase C (PKC)/nuclear factor *κ*B (NF-*κ*B) signaling pathways, resulting in vascular endothelial injury and apoptosis (41). Moreover, HDL-C exhibits antioxidant properties that alleviate lipid peroxidation and oxidative stress through reverse cholesterol transport and the activity of antioxidant enzymes (42). Increased NHHR correlates with reduced HDL-C levels, compromising cellular defense mechanisms against oxidative stress and increasing susceptibility to pro-inflammatory cytokines, such as including interleukin-6 (IL-6) and tumor necrosis factor-alpha (TNF-α), thereby exacerbating cellular inflammation and injury (43, 44).

An ideal biomarker should exhibit both high sensitivity and specificity. In this study, ROC curve analysis was conducted to evaluate the predictive values of NHHR, HDL-C, TG, and the TyG index. The AUC values indicated that HDL-C and TG exhibited relatively high sensitivity but relatively low specificity, whereas NHHR and TyG index exhibited the opposite characteristics. Notably, NHHR achieved the highest AUC value, along with moderate sensitivity (50.88%) and high specificity (87.04%), suggesting its potential as a promising biomarker for diagnosing and predicting the prognosis of RVO patients. Hypertension was identified as the strongest risk factor for any type of RVO, with a meta-odds ratio (OR) of 2.82 (95%CI: 2.12–3.75) (2). Age is a significant risk factor, as the incidence increases markedly in individuals aged 50 years and older (3); however, RVO can also occur in younger patients under the age of 40 (4) or under 55 years old (45). Additionally, BRVO was more prevalent in women (54.5%), while CRVO was more common in men (50.4%) (46). Therefore, we recommend that hypertensive individuals aged 40 years and older include liver function tests in their routine physical examinations, with particular attention to measuring total cholesterol and HDL-C levels. Blood HDL-C and total cholesterol concentrations were quantified utilizing enzymatic methods (47). The NHHR was calculated as (Total cholesterol—HDL-C)/HDL-C (47).

Several limitations should be acknowledged in the present study. First, the relatively small sample size might limit the generalizability of our findings to a broader population. Conducting longitudinal studies is exceedingly pivotal to confirm the association between NHHR and RVO. Second, the case-control design does not permit causal inference between exposure and disease; thus, it remains unknown whether the elevated NHHR is a direct cause or merely a reflection of other underlying factors associated with RVO. Third, as the samples were recruited from a single center, the results primarily reflect the biological characteristics of RVO in the local ethnic population. Due to data constraints, the applicability of these findings to patients from other centers requires further validation. Fourth, although several covariates were included, not all potential confounding variables related to NHHR were accounted for in this study. While adjustments were made for possible confounding factors, the influence of unmeasured systemic inflammation variables, such as the systemic immune-inflammation index (SII) and systemic inflammatory response index (SIRI), cannot be entirely excluded. Fifth, we recommend conducting large-scale multi-center cohort studies using standardized fundus photography instruments and ophthalmological examination protocols to further validate the association between NHHR and RVO.

## Conclusion

In conclusion, our findings indicate that NHHR is significantly elevated in patients with RVO. An NHHR ratio greater than 2.37 demonstrates 50.88% sensitivity and 87.04% specificity in predicting RVO. Thus, we propose that NHHR may serve as a valuable, cost-effective, practical, and easily obtainable biomarker for assessing RVO development. Nevertheless, further investigations are necessary to elucidate the diagnostic and prognostic utility of NHHR in RVO across larger and more diverse populations.

## Data Availability

The original contributions presented in the study are included in the article/Supplementary Material, further inquiries can be directed to the corresponding authors.
